# Neurological, Psychiatric, and Multisystemic Involvement of Fragile X Syndrome Along With Its Pathophysiology, Methods of Screening, and Current Treatment Modalities

**DOI:** 10.7759/cureus.35505

**Published:** 2023-02-26

**Authors:** Raunak Ranjan, Saumya Jha, Priyadarshi Prajjwal, Ansh Chaudhary, Pragya Dudeja, Neel Vora, Mohammed A Mateen, Mohammed A Yousuf, Bhupendra Chaudhary

**Affiliations:** 1 Internal Medicine, Bharati Vidyapeeth University Medical College, Pune, IND; 2 Neurology, Bharati Vidyapeeth University Medical College, Pune, IND; 3 Internal Medicine, Kasturba Medical College, Manipal, Manipal, IND; 4 Internal Medicine, B.J. (Byramjee Jeejeebhoy) Medical College, Ahmedabad, IND; 5 Internal Medicine, Shadan Institute of Medical Sciences Teaching Hospital and Research Centre, Hyderabad, IND; 6 Neuroscience, Jaswant Rai Speciality Hospital, Meerut, IND

**Keywords:** autism spectrum disorder (asd), fragile x mental retardation 1, fragile x syndrome, intellectual disability (id), autism, connective tissue disorder(ctd), neuropsychiatric, cgg repeats, fmr1 gene

## Abstract

Fragile X syndrome (FXS) is a hereditary disease that predominantly leads to intellectual disability (ID) in boys. It is the second prominent cause of ID, which manifests as a result of the atypical development of the cytosine-guanine-guanine (CGG) region. This irregular extension of the CGG region gives rise to methylation and silencing of the fragile X mental retardation 1 (*FMR1*) gene, causing a loss of the fragile X mental retardation 1 protein (FMRP). This reduction or loss of FMRP is the main cause of ID. It has a multisystemic involvement showing neuropsychiatric features such as ID, speech and language delay, autism spectrum disorder, sensory hyperarousal, social anxiety, abnormal eye contact, shyness, and aggressive behaviour. It is also known to cause musculoskeletal symptoms, ocular symptoms, cardiac abnormalities, and gastrointestinal symptoms. The management is challenging, and there is no known cure for the disease; hence an early diagnosis of the condition is needed through prenatal screening offered to couples with familial history of ID before conception. The management rests on non-pharmacological modalities, including applied behaviour analysis, physical therapy, occupational therapy, speech-language therapy, and pharmacologic management through symptomatic treatment of comorbid behaviours and psychiatric problems and some forms of targeted therapy.

## Introduction and background

Bell and Martin, in 1943, put forward a noteworthy investigation in the field of genetics by recognising Fragile X syndrome (FXS) as a genetic disorder that is an underlying cause of intellectual disability (ID). FXS, also called Martin-Bell syndrome, is prompted by a repeated sequence of three nucleotides on the X chromosome, known as a trinucleotide repeat [1]. Further research in 1991 established the association of the specific fragile region on the X chromosome with ID. Lubs initially made this finding in 1969 by observing this relationship in three families [2,3]. FXS, a genetic disorder known to cause ID, results from a trinucleotide repeat on the X chromosome and is now regarded as the principal hereditary cause of ID in boys, following Down syndrome accounting for 2.4% of all ID cases [4,5]. According to estimates, there are about 1:5000 males, and 1:6000 females affected globally. The disease has multisystemic implications and is known to cause neuropsychiatric, ocular, and gastric symptoms. In addition, it shows characteristics similar to the connective tissue disorder spectrum.

Methods 

**Figure 1:**
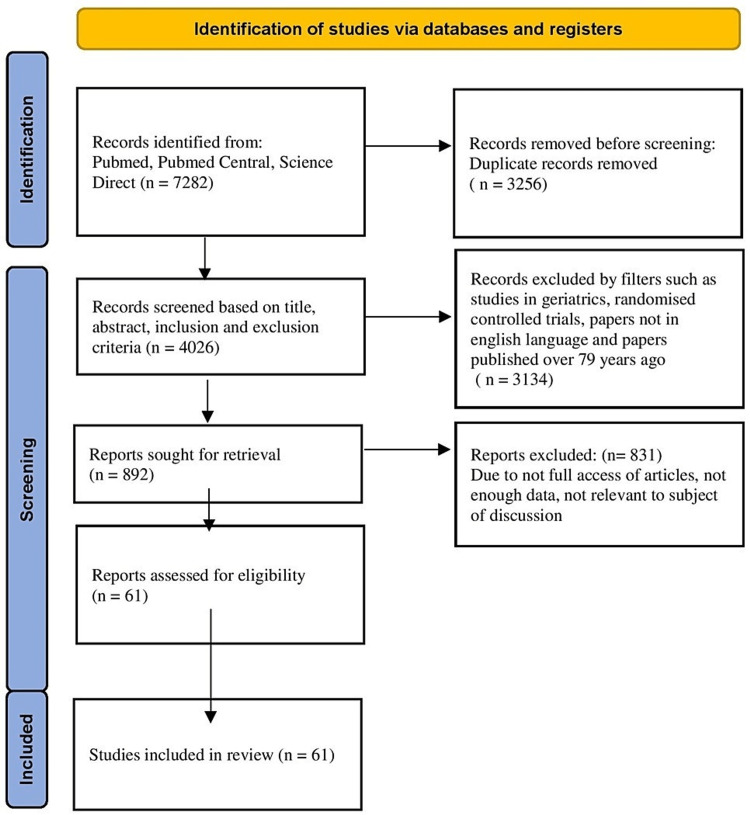
PRISMA Flow Diagram PRISMA: Preferred Reporting Items for Systematic Reviews and Meta-Analyses

Preferred Reporting Items for Systematic Reviews and Meta-Analyses (PRISMA) guidelines guided establishing this systematic review to optimize its rigour and comprehensiveness (Figure 1).

Study Search and Criteria of Selection

Searches were conducted on PubMed Central, PubMed, and Science Direct libraries of articles published between January 1943 and January 2022, and relevant references were retrieved. The search terms "Fragile X mental retardation 1 (FMR1) gene", "Cytosine-guanine-guanine (CGG) repeats", "Autism spectrum disorder", and "Fragile X syndrome (FXS)" were used, and the obtained studies were assessed for suitability. Articles written in the English language, research focusing on children, adolescents, and adults, and literature pertaining to the topic of discussion were taken into consideration. Relevant studies were then broadly investigated regarding the research in question using title and abstract. Geriatric-based research, articles in languages other than English, papers published over 79 years ago, and unrelated studies were excluded.

## Review

Pathophysiology

Thefragile X mental retardation 1 (*FMR1) *gene is located on Xq27.3 and undergoes an increase of CGG repeats (>200), which is the cause of FXS. The aberrant CGG expansion causes the *FMR1* gee to be methylated and transcriptionally silenced, which causes fragile X mental retardation 1 protein (FMRP) to be reduced or lost. FMRP is a protein that travels to and from the nucleus and may be involved in the nuclear export of messenger ribonucleic acids (mRNAs). Immature axons, growth cones, dendritic spines, and mature dendrites, all contain FMRP. Arc and MAP1b are two proteins whose translation is structured by FMRP, which are known to regulate α-amino-3-hydroxy-5-methyl-4-isoxazole propionic acid (AMPA) receptor endocytosis and, thereby, synaptic function. Due to FMRP deficiency, there is a loss in synaptic growth and plasticity. Formation of long, thin, juvenile dendritic spines occurs, which can impair cognitive and learning abilities [6]. The *FMR1* gene can be classified into one of four groups (determined by the number and redundancy of CGG): The most frequent is 6-44 CGG repeats in humans; 45-54 CGG repeats are called intermediate alleles or "grey zone"; 55-200 CGG repeats are premutation alleles; and >200 CGG repeats are full-length mutations [7]. During the second and third years of life, developmental delays become more pronounced, whereas speech delays are noticed in the first year of life.

Gender affects the clinical representation of FXS. Due to compensated amplification of the normal X chromosome, males are more susceptible than females, who typically exhibit a less severe phenotype [8]. FXS is often underdiagnosed in females, with only 25-30% accounting for ID. Around 30% of females with FXS present with borderline intelligence quotients (IQs); however, those with normal IQs may encounter emotional imbalance or learning disorders [8].

FXS presents several neurobehavioural disorders. More than 80% of people with FXS show recognizable physical characteristics, such as an elongated face, large or prominent ears, a high palate, hypermobile joints, and macroorchidism (during puberty or later) [9-10]. About 80-95% of adults have pubertal macroorchidism, which is thought to be a defining characteristic of the disorder. The most prominent FXS symptoms include specific learning disability (SLD), autism spectrum disorder (ASD), ID, shyness, social anxiety, sensory hyperarousal, abnormal eye contact, aggressive behaviour, sleep issues, attention deficit hyperactivity disorder (ADHD), hand flapping, seizures, repetitive behaviours, and even obsessive-compulsive disorder (OCD). Approximately 90% of males diagnosed with FXS showed signs of developmental delays, and around 50-60% are also diagnosed with ASD [11-14]. Seizures occur in 15-20% of FXS patients, notably in those who had ASD [15]. Additionally, obesity and digestive disorders, including gastroesophageal reflux, affect nearly 30% of patients [16].

A true syndrome: multisystem involvement

Neurological and Psychiatric Involvement 

Deferral in psychomotor development is regarded as the first indication of impending ID in school-going children, and it is a relatively early finding. By the age of six months, all areas of development, including visual reception, fine motor, expressive communication, and receptive communication in FXS-presenting infants differ from those of the conventional cohort [17]. In individuals with FXS, ADHD is regarded as a common comorbidity, with over 50% of male FXS patients experiencing it at some point in their lives. The occurrence of ADHD symptoms in this population ranges from 54% to 59%, being higher than that observed in individuals with a single ID or other neuropsychiatric disorders [18]. About 12-23% of FXS participants highlight the symptomatology for ADHD [19,20]. Pervasive developmental disorder (PDD) [21], stereotypies [22], sleep issues [23], specific or social phobias, selective mutism [24], compulsive and ritualistic behaviour, restricted interests [22], aggressiveness, and self-injurious behaviour [25] are additional neuropsychiatric conditions. A recent study conducted by Wheeler et al. extensively analysed this aspect and found that 90% of individuals with FXS, whether male or female, reported engaging in at least one aggressive act in the past year [26]. Sleep disturbances, which include trouble with frequent nighttime awakenings, falling asleep, and early morning awakenings, impact 30% of FXS youngsters of both sexes [23]. The hallmark of the syndrome is undoubtedly the neurologic/neuropsychiatric presentation. Still, FXS also relates to several medical issues that may or may not be present; when they are present, they can exacerbate the phenotype of patients and make clinical management more challenging.

It has been evident that FXS and connective tissue disorder (CTD) spectra share some characteristics since the ailment was first reported. The prevalence of connective tissue symptoms suggests the existence of an abnormality in the underlying connective tissue, similar to what is observed in CTDs, even if a specific connective tissue abnormality such as Ehlers-Danlos syndrome (EDS) or Marfan syndrome (MFS) has not been demonstrated yet. Skin can be soft [27-29], and joint hypermobility, which primarily affects tiny joints (mainly metacarpal-phalangeal joints), exists in nearly half of the patients [29]. Flat feet, a high-arched palate, scoliosis, and pectus excavatum are examples of skeletal symptoms [30].

In FXS patients, the heart is also influenced by heightened connective tissue fragility, which leads to cardiac aberrations comparable to those with CTDs. Aortic root dilatation, presenting in about one-fourth (25%) of the patients, and mitral valve prolapse (MVP), affecting 3-50% of patients, are common conclusions [31]. Additional findings include parasympathetic vagal tone reduction and hyperarousal (i.e., a higher heart rate). Adult FXS patients (older than 40 years) frequently experience the same cardiovascular issues as the general population in their age group, including cardiac rhythm difficulties (24.2%) and hypertension (24.2%) [32].

The functioning of the gastrointestinal (GI) system in individuals with FXS has yet to be thoroughly investigated. Hypotonia and connective tissue anomalies may be responsible for some gastrointestinal issues associated with this syndrome, including gastro-oesophageal reflux, loose stools, and constipation. About 30.6% of FXS-presenting individuals aged 40-71 report digestive issues [32]. Ocular signs are reported in at least 25% of children with FXS and a higher proportion of adults with FXS. Among them are strabismus 8-40%, and refractive errors 17-59% (commonly, astigmatisms and hyperopia, while sporadic events of myopia are also reported), whereas nystagmus is rarely spotted (5-13%). Other noticeable ocular characteristics include convergence insufficiency and palpebral ptosis [33-36]. Recurrent otitis media in children with FXS often results in conductive hearing loss [37]. Since these individuals already have limited expressive language abilities, it is crucial that any potential otologic issues are treated urgently to prevent impeding their ability to speak more clearly [38].

Differential diagnoses 

Sotos Syndrome

Sotos syndrome is a genetic disorder that presents with abnormal growth developments such as peculiar facial characteristics, overgrowth in childhood, and compromised development of cognitive and motor abilities. Other associated attributes include immoderate height and an excessively large cranial circumference. Furthermore, individuals with Sotos syndrome may exhibit phenotypic similarities with FXS-afflicted patients, such as ASD, ADHD, phobias, obsessive-compulsive behaviours, impulsive behaviours, and tantrums.

Prader-Willi Syndrome

Prader-Willi syndrome (PWS) is a genetic disorder caused by a deletion or abnormality in a specific region of chromosome 15. It is a rare condition, affecting approximately one in 10,000 to 30,000 individuals worldwide. The symptoms of PWS can be physical, cognitive, and behavioural, and they can vary widely from person to person.

Klinefelter Syndrome

Klinefelter syndrome is a genetic disorder that affects males and is caused by an extra X chromosome. It is estimated to affect approximately one in 500 to 1,000 male births. Individuals with Klinefelter syndrome may experience various physical, cognitive, and behavioural symptoms, including infertility, reduced muscle mass, increased body fat, and learning disabilities. Treatment for Klinefelter syndrome typically involves testosterone replacement therapy and may include educational support for learning challenges.

Rett Syndrome and Angelman Syndrome

In the differential diagnosis of FXS, Rett syndrome and Angelman syndrome must be taken into account, irrespective of the fact that their clinical presentation may differ from that of FXS. Common characteristics between these syndromes and FXS include ID, language impairments, and autistic behaviour. Array comparative genomic hybridisation (CGH) may be carried out to rule out the possibility of cytogenetic rearrangements causing ID. When genetic testing proves inconclusive, isolated ID, autism, or ADHD should be deliberated as probable diagnoses [39].

Screening and diagnosis 

Prenatal FMR1 testing

As mentioned above, FXS molecular testing is often conducted postnatally on peripheral blood lymphocytes (when the necessary clinical features are present). Furthermore, it is possible to perform prenatal testing for FXS by using long-range-polymerase chain reaction (LR-PCR) techniques on DNA obtained from amniocytes or chorionic villi. According to the latest guidelines from the American College of Medical Genetics (ACMGG) and the American Congress of Obstetricians and Gynecologists (ACOG), couples who have a personal or family history of any of the following should be provided *FMR1 *prenatal testing: unexplained ID or developmental delay, FXS- or FX-related disorders, isolated cognitive impairment, isolated cerebellar ataxia with tremor, idiopathic familiar primary ovarian insufficiency or elevated follicle-stimulating hormone (FSH) at age <40 years, and autism.

Many geneticists recommend that antenatal screening for FXS be made available to all women who desire it, regardless of their personal or family history, due to the high FXS incidences in public [40]. The test should be offered to couples who desire FXS screening before conception, giving the pair a chance to make informed reproductive decisions. The parents must also provide pertinent information regarding their health.

Advanced Diagnostic Methods

**Figure 2:**
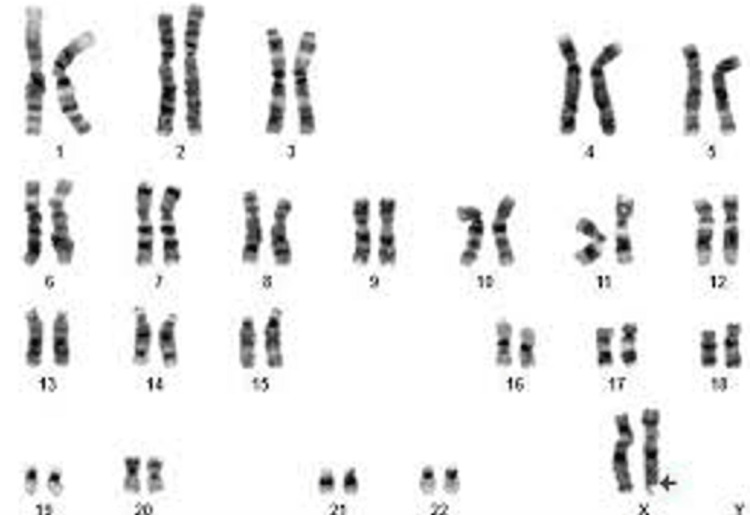
G-banding karyotype (≥500 bands) showing apparently a normal X and fragile X chromosome (Xq27.3) Image Source: Vafaeie et al., 2021 [41]

Over time, the diagnosis of FXS has changed. It was initially based on the cytogenetic analysis of fragile X (FRAXA)'s existence in peripheral blood lymphocytes (G-banding) (Figure 2). However, it was restricted due to issues including the procedure's length, difficulty in interpretation, and requirement for specific technical abilities.

To overcome the constraints of previously practised testing procedures, fluorescence in situ hybridisation (FISH) has been established as an alternate method for distinguishing FXS. This method uses DNA probes labelled with fluorescent dyes to identify the exact position of genetic material, thereby increasing the detection rate of FXS. The use of DNA Southern blot analysis for *FMR1 *gene testing has been recognized as the ideal method for cytogenetic analyses, as it helps identify all *FMR1* alleles, including normal, permutation (PM), and full mutation (FM), as well as determining the methylation status of the *FMR1* promoter region. However, this technique is likewise complex and resource-intensive as its predecessors, rendering it a complicated and costly procedure.

The current standard for FXS molecular analysis is PCR combined with Southern blot analysis [42,43]. Methylation status and the number of CGG repeats can be assessed with the help of a standard PCR and Southern blot analysis. Calculating the number of CGG repeats on the X chromosome enables precise FXS risk assessment and offers FXS families information about their reproductive possibilities. It is important to note that relying solely on the amount of CGG repeats will pick up fewer than 1% of FXS produced by deletions or missense mutations in the *FMR1* gene. Potential "non-CGG repeat" causes of FXS could be found using precise measurements of the FMRP level and sequencing of the *FMR1* gene [44]. However, it could only identify alleles with up to 160 and 300 repetitions in females and 300 repeats in males, respectively, failing to detect massive CGG expansions [45,46].

The triplet primed PCR (TP-PCR) is a relatively latest method for detecting *FMR1* alleles, which involves the use of three primers, including a forward primer situated in the upstream CGG region, a second primer that covers both the CGG repeat and the adjacent unique sequence, and a third primer that is complementary to the *FMR1* triplet repeat region. Using TP-PCR, it is possible to amplify the full-length *FMR1* allele and the CGG triplets together in a single PCR reaction. The number of CGG repeats is then assessed by evaluating the size of the PCR products through capillary electrophoresis. TP-PCR has gained popularity in being regarded as the most accurate and reliable method for the diagnosis of FXS. Furthermore, the methylation-sensitive long-range PCR (MS-LR-PCR) kit can be used to perform CGG methylation testing as a follow-up analysis in the diagnostic process to investigate the silencing of FMRP [47].

Treatment

Treatment can be classified into three categories.

Non Pharmacological 

**Figure 3:**
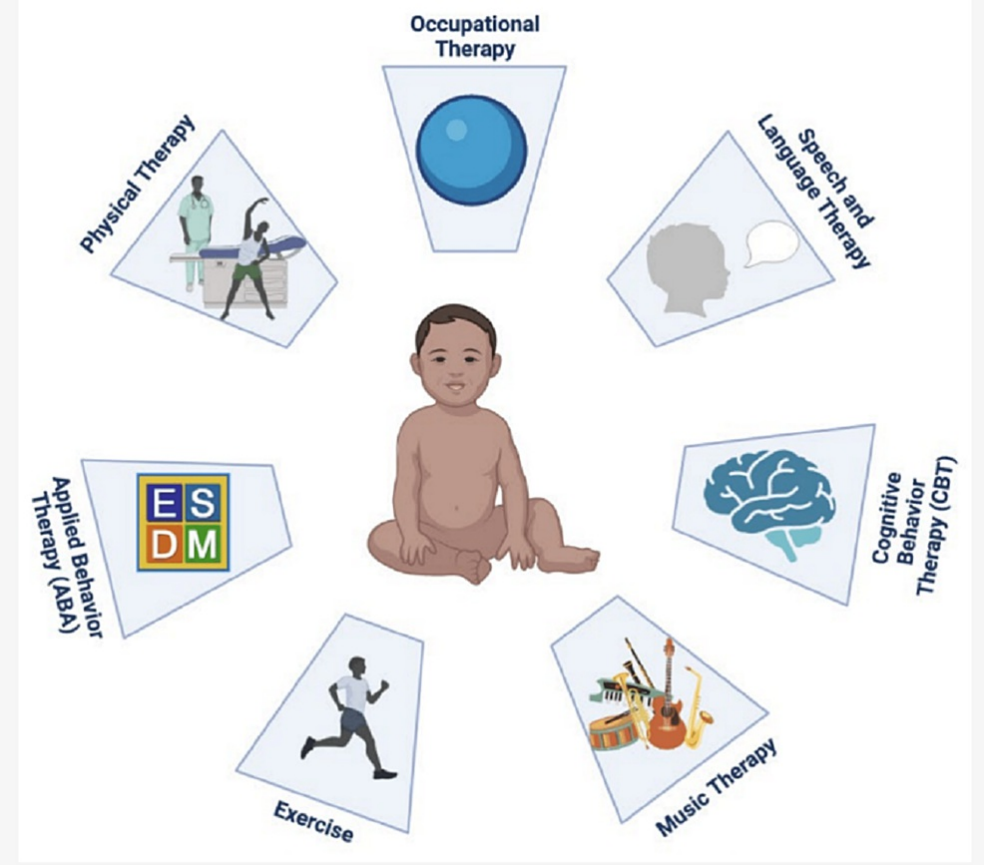
An overview of non-pharmacological treatment modalities in FXS Image Source: Protic et al., 2022 [49]

Physical therapy (PT), occupational therapy (OT), speech-language therapy (SLT), and ABA are essential non-pharmacological interventions (Figure 3) for FXS in dealing with motor and speech-language problems and social communication skills in those with comorbid ASD. Behavioural therapy (parent training) can assist children and teens with behavioural problems like aggression, hyperactivity, and tantrums. Cognitive behavioural therapy (CBT) can play a role in facilitating women and high-functioning males with anxiety, ADHD, social challenges, and depression through individual therapy [48,49]. SLT and OT are the two most preferred interventions for FXS-afflicted children [50]. SLT is widely used to treat FXS children and address their delayed communicative development.

For FXS individuals, OT tackles the sensory integration challenges common among the population. OT also aims at improving sensory processing, sensorimotor abilities, and play engagement for young children while encouraging independence and job readiness for teens. Research has revealed that vocational training programs emphasizing life skills such as community involvement, computer skills, financial management, and interpersonal interactions can provide long-term benefits by offering improved satisfaction and stable employment [51-53]. Parent-implemented language intervention (PILI) strives to enhance communication and language skills by teaching parents how to incorporate responsive skills in interacting with their child. It attempts to help promote language development. Such intervention can be adopted in the home environment and is designed to be integrated into the child's daily activities. PILI effectively improves language skills in children with FXS and is considered a promising approach for addressing the language challenges that often accompany the disorder [54].

ABA is a different intervention used with FXS kids, notably those with ASD. The premise underlying ABA is that positive actions should be reinforced. The Early Start Denver Model (ESDM) is a therapeutic approach comprising principles of ABA, focusing on refining language skills and social interactions in young children between one and three years of age in a realistic home setting. PT is also a generally adopted intervention for children with FXS [50]. FXS patients experiencing difficulty with walking and balance may receive recommendations from physical therapists for assistive devices, such as strollers for stability or orthotics to correct foot pronation [55]. Physical activity is linked to enhanced attention and cognitive control in developing children [56]. Exercise causes a rise in brain-derived neurotrophic factor (BDNF), crucial for brain development and neuroplasticity, which may help explain this phenomenon [57]. Elevated anxiety levels in kids with FXS can be treated with CBT, a talk therapy that has been proven useful in regulating emotions. Music therapy has also increased self-expression and communication abilities in children with FXS [58].

Psychopharmacological

Anxiety is a common issue among those with FXS. Sertraline, a selective serotonin reuptake inhibitor (SSRI), is an efficacious treatment modality for this condition. SSRIs prevent the presynaptic reuptake of serotonin, thereby increasing serotonin levels in the synapse, which play a crucial role in regulating mood in the central nervous system. Another antidepressant known to elevate noradrenergic and dopaminergic neurotransmission is bupropion, which blocks reuptake [59].

People with FXS may exhibit aggressive behaviour, involve in self-harm, and display violent outbursts or "meltdowns," particularly during their teenage years [60]. Risperidone and aripiprazole are two types of atypical antipsychotics that can effectively address symptoms of aggression and meltdowns, which often accompany anxiety in FXS. They work by activating serotonergic and dopaminergic receptors in the brain.

ADHD is categorized by continuous episodes of distractibility, hyperactivity, and/or impulsivity that are more different or upsetting than children of the same age and trigger complications in multiple settings [61]. For children aged six years and above with ADHD, stimulant medication is the first line of treatment. These drugs enhance the levels of norepinephrine and dopamine in the prefrontal cortex, which are vital for improving task motivation, attention, and impulse control. Moreover, clonidine and guanfacine (alpha 2-adrenergic receptor agonists) may also be advantageous in handling ADHD symptoms [62]. Alternative treatments may be considered for individuals who either do not respond to or cannot tolerate stimulant medications, such as young children under the age of years. Clonidine and guanfacine (alpha 2-adrenergic receptor agonists) can improve attention and provide a comforting effect on hyperarousal by enhancing norepinephrine levels in the prefrontal cortex. It can be particularly helpful for those with FXS.

Sleep issues are predominant among FXS sufferers. It is characterized by sleep difficulty ranging from 27% to 77% in individuals with FXS [63]. Melatonin is regarded as the most effective treatment for this illness. The hormone is released from a pineal gland under typical environments at night, accelerates the sleeping process, and increases the length of nighttime sleep. Moreover, it exhibits neural plasticity and anti-oxidative properties, which may aid memory and learning [64]. Nevertheless, a few side effects of melatonin have also been reported, such as daytime sleepiness and nausea.

In those with FXS, seizures are effectively managed by a single anticonvulsant. Several anticonvulsant drugs are used in the course of treatment. For controllable side effects, levetiracetam and oxcarbazepine are regularly used as first-choice treatments. Substitute medications like valproic acid and lamotrigine may also be effective, particularly for those with generalized seizures. However, due to the severity of their side effects, phenytoin, phenobarbital, and gabapentin should be avoided.

Targeted Therapy

Over the past decade, much effort has been devoted to introducing specific treatments for FXS. Currently, two approaches are being explored as potential major treatments for FXS: Reactivating the damaged gene is one option, and making up for FMRP's absence is another. The hypothesis describing how excessive translation in FXS, which is typically triggered by stimulation of the mGluR or another Gq-coupled receptor (when FMRP is absent), leads to cognitive and behavioural manifestations and has provided multiple potential sites for targeted therapy (Table 1 and Table 2). However, these techniques are constantly being developed, and there is currently no known cure for FXS.

**Table 1:** Potential sites for targeted therapy of fragile X syndrome KO: knock-out; FXS: fragile X syndrome; mEPSP; miniature excitatory postsynaptic potentials; LTP: long-term potentiation; ERK: extracellular signal-regulated kinase Information from: Berry-Kravis et al., 2011 [65]

Reversal of Phenotypic features			Progress in Translation
dFMR1 mutant fly		*FMR1* KO mouse	FXS-carrying humans
Halt the external pathway of translational signalling:			
mGluR5 inhibition; Fenobam, STX107, MPEP, RO4917523, AFQ056; Inhibition of mGluR5 by lowering mGluR5 receptors genetically	Courtship behaviour: Instant recall and temporary memory; Survival on glutamate-rich food; Odour-shock memory; Body revealing mushroom appearance	Rupture of epileptiform; Hyperactivity in an open-field environment; Convulsive seizures produced by a high-frequency sound; dendritic spine morphologya; Prepulse inhibition; Amygdala mEPSP frequency; Marble burying​​​​; Plasticity in the visual cortex; Extinction of passive avoidance; Density in dendritic spine; Abnormally high protein synthesis	STX107-Phase I completed; Fenobam- Phase IIa (single-dose); PPI alleviated, and anxiety was reduced; AFQ056 - Phase IIb; improvement in patients exhibiting complete methylation; phase III trial is commenced; RO4917523 - phase II trial underway
Halt the internal pathway of translational signalling:			
GSK3β inhibition using AR-A014418 or SB-216763; PAK inhibition through genetic reduction of PAK; PI3K inhibition using LY294002; ERK/MEK inhibition using SL327, -Lithium is used to inhibit GSK3β and reduce the short-term turnover of P.		-Dendritic spine morphology; Open-field hyperactivity; Audiogenic seizures; Learning and anxiety deficits in the elevated plus maze; Elevated zero mazes; Passive avoidance; Social interaction deficit; Anxiety during social interaction; Impairment in cortical LTP; Behavioral redundancy	Behavioural improvement was observed in an open-label trial, with improvement in some adaptive skills and verbal memory; Normalisation of ERK biomarker

**Table 2:** Potential sites for targeted therapy of fragile X syndrome KO: knock-out; FXS: fragile X syndrome; STEP: striatal-enriched protein tyrosine phosphatase; APP: amyloid precursor protein; MMP9: matrix metalloproteinase 9; AMPA: α-amino-3-hydroxy-5-methyl-4-isoxazolepropionic acid; FMRP: fragile X mental retardation 1 protein; BDNF: brain-derived neurotrophic factor; TBS: Townes–Brocks syndrome; LTP: long-term potentiation; GABA: gamma-aminobutyric acid; NMDA: N-methyl-D-aspartate, ERK: extracellular signal-regulated kinase Information from: Berry-Kravis et al., 2011 [65].

Reversal of Phenotypic Features			Progress in Translation
dFMR1 mutant fly		*FMR1* KO mouse	FXS-carrying humans
Prevent the activity of specific proteins regulated by FMRP:			
Inhibit STEP through genetic reduction of STEP; Inhibit APP/Aβ through antibody or genetic reduction of APP; Inhibit MMP9 with minocycline	Anxiety behaviour in the elevated plus maze; Inquisitive behaviour in Y maze -dendritic spine morphology; Internalisation of AMPA receptor; Convulsive seizures produced by a high-frequency sound; Open-field hyperactivity	Behaviour improved (small open-label trial)	Inhibit STEP through genetic reduction of STEP; Inhibit APP/Aβ through antibody or genetic reduction of APP; Inhibit MMP9 with minocycline
Stimulate cell-surface AMPA receptors:			
Ampakines (CX516, CX614)		The increase in BDNF levels caused by CX614 leads to reversing impairments in TBS-LTP in the hippocampus.	Phase II trial of CX516 showed no significant impact but may have helped those on antipsychotics (due to low dose).
Other proteins/synaptic receptors:			
GABA-B agonists (baclofen, R- baclofen); NMDA Antagonists -Anticholinesterse (donepezil); GABA-A agonists (ganaxolone)	The ability to survive on food containing glutamate and memory impairments.	Audiogenic seizures are caused by sound, increased activity in open spaces, and burying marbles.	Phase II trial of R-baclofen shows improvement in overall function and social and language abilities, particularly in those with more severe social impairments; An open-label trial demonstrated improvement in behaviour and social skills; A small open-label trial of Memantine also showed positive results; An open-label trial of acamprosate manifested improvement in language; An open-label trial of riluzole normalised ERK; overall improvement was negligible.

Limitations of the study

Our thorough investigation has a few limitations that need to be acknowledged and recorded. Limited research and clinical trial data hinder the development of effective treatments for targeted therapy. Since this is a fairly new approach and many studies are still being conducted using it, there is a shortage of documentation available. Another key aspect to consider when selecting the studies was free full-text availability. Last but not least, there is a risk of information bias because of increased global interest in the study's subject.

## Conclusions

FXS is a true syndrome in that it affects multiple body systems and presents with varied clinical features. FXS poses a disorder with a huge burden on the child and his family, both socially and financially. Due to the majority of its clinical features being in the same domain as diseases such as Angelman syndrome and Rett syndrome, Klinefelter syndrome, Prader-Willi syndrome, and Sotos syndrome, a clinician should always keep his mind open to the possibility of FXS in an intellectually disabled child. Over the years, numerous techniques for prenatal testing and early diagnosis have been developed. Non-pharmacological treatments addressing gross and fine motor, speech-language, and social-communication deficiencies have proved effective. With ongoing clinical trials for targeted management through mGluR5 inhibition, GSK3, and PAK inhibition by genetic reduction, an effort towards curing the disease is being sought to reduce the disease burden.
